# End of life care in paediatric settings: UK national survey

**DOI:** 10.1136/spcare-2023-004673

**Published:** 2024-11-28

**Authors:** Andre Bedendo, Andrew Papworth, Bryony Beresford, Bob Phillips, Chakrapani Vasudevan, Gabriella Lake Walker, Helen Weatherly, Richard Feltbower, Sebastian Hinde, Catherine Elizabeth Hewitt, Fliss Murtagh, Jane Noyes, Julia Hackett, Richard Hain, Sam Oddie, Gayathri Subramanian, Andrew Haynes, Lorna Fraser

**Affiliations:** 1 Department of Health Sciences, University of York, Heslington,York, UK; 2 Social Policy Research Unit, School for Business and Society, University of York, York, UK; 3 Centre for Reviews and Dissemination, University of York, York, UK; 4 Bradford Hospitals National Health Service Trust, Bradford, Bradford, UK; 5 Parent Advisory Panel Member, Department of Health Sciences, University of York, Heslington, York, UK; 6 Centre for Health Economics, University of York, Heslington, York, UK; 7 Leeds Institute for Data Analytics, School of Medicine, University of Leeds, Leed, UK; 8 York Trials Unit, University of York, Heslington, York, UK; 9 Wolfson Palliative Care Research Centre, Hull York Medical School, University of Hull, Hull, UK; 10 School of Health and Medical Sciences, Bangor University, Fron Heulog, Bangor, UK; 11 All-Wales Paediatric Palliative Care Network, Cardiff and Vale University Health Board, Cardiff, UK; 12 College of Human and Health Sciences, Swansea University, Swansea, UK; 13 Manchester University National Health Service Foundation Trust, Manchester, UK; 14 Cicely Saunders Institute, Faculty of Nursing, Midwifery and Palliative Care, King’s College London, London, UK

**Keywords:** Paediatrics, Service evaluation, Terminal care

## Abstract

**Objectives:**

To describe end of life care in settings where, in the UK, most children die; to explore commonalities and differences within and between settings; and to test whether there are distinct, alternative models of end of life care.

**Methods:**

An online survey of UK neonatal units (NNUs), paediatric intensive care units (PICUs) and children/young people’s cancer principal treatment centres (PTCs) collected data on aspects of service organisation, delivery and practice relevant to end of life outcomes or experiences (referred to as the core elements of end of life care) across three domains: care of the child, care of the parent and bereavement care.

**Results:**

91 units/centres returned a survey (37% response rate). There was variation within and between settings in terms of whether and how core elements of end of life care were provided. PTCs were more likely than NNUs and PICUs to have palliative care expertise strongly embedded in the multidisciplinary team (MDT), and to have the widest range of clinical and non-clinical professions represented in the MDT. However, bereavement care was more limited. Many settings were limited in the practical and psychosocial-spiritual care and support available to parents.

**Conclusions:**

Children at end of life, and families, experience differences in care that evidence indicates matter to them and impact outcomes. Some differences appear to be related to the type of setting. Subsequent stages of this research (the ENHANCE study) will investigate the relative contribution of these core elements of end of life care to child/parent outcomes and experiences.

**WHAT IS ALREADY KNOWN ON THIS TOPIC**

- There is little systematic evidence in the UK on how end of life care is organised and practised in services most likely to be caring for babies, children and young people with life-threatening or life-shortening conditions.

**WHAT THIS STUDY ADDS**

- Neonatal and paediatric services vary in how they manage and deliver end of life care. This is partly determined by type of setting (ie, intensive care vs oncology)

**HOW THIS STUDY MIGHT AFFECT RESEARCH, PRACTICE OR POLICY**

- Later phases of the study are investigating the impacts of these differences on child and parent outcomes and experiences. When available, such evidence should be used to inform funding/commissioning decisions and efforts to develop or improve services.

## Introduction

 Advances in public health and medical treatment have resulted in marked reductions in childhood mortality but around 4200 babies and children (0–18 years) still die in the UK each year, with around half having an existing life-threatening or life-shortening condition.^1^ End of life care is defined by the National Health Service (NHS) in England as the care needed when a patient is approaching the end of their life and may require the involvement of multiple professions and hospital-based and community-based services.^2^ In an effort to secure consistency in how the term ‘approaching end of life’ is applied, and more tightly define the period in the condition trajectory it refers to, use of the ‘surprise question’ (Would you be surprised if this patient died in the next 12 months?) was suggested. This has been found to be effective and has gained significant traction in adult and geriatric medicine,^3 4^ and more recently paediatrics.^5^ However, overall, end of life is harder to predict in the paediatric population compared with adults and older people. This increases the likelihood of inadequate planning for end of life and reduced choices available with respect to place of care and death. Paediatric intensive care, neonatal units and children’s cancer services are the most common settings where babies and children die, or from which they are transferred home or to a hospice to die.

As well as limitations in existing evidence on symptom management at end of life,^6 7^ there is also uncertainty about what matters to children and parents in terms of the way end of life care is organised and delivered, and how this might affect ‘quality of death', bereavement outcomes, and patient/parent experience.^6^ This lack of evidence hinders decision-making around commissioning and service improvement or development.

This paper reports the first stage of the ENHANCE study.^8^ Its overall aim is to investigate whether the way end of life care is provided (ie, models of care^9^) in UK neonatal and paediatric intensive care units, and children’s cancer services, affects end of life outcomes and experiences. A necessary first stage of the study was to identify and describe the different models of end of life care currently operating in these settings, as defined by a number of aspects of service organisation and delivery and the non-clinical care and support available.

## Methods

### Study design and settings

Cross-sectional, online survey of clinical/service leads of clinical settings in the UK most likely to be caring for babies, children and young people at end of life, namely:

- Neonatal units (NNU): comprising short-term, low-dependency units (special care baby units (SCBU)), high-dependency units (local neonatal units (LNU)) and complex care neonatal intensive care units (NICU) (~1100 deaths per year)^10^
- Paediatric intensive care units (PICUs) (~700 deaths per year)^11^
- Children and young people (CYP) and teenage and young adult (TYA) principal treatment centres (PTCs) (~350 deaths per year)^12^

For ease of reading, for the remainder of this article, we use the word ‘unit’ to collectively refer to units and centres.

### Sampling and recruitment

Clinical leads/directors of all UK NNUs (n=181), PICUs (n=28) and PTCs (n=38) were invited to take part in the survey or delegate completion to the unit’s palliative care lead or other member of staff, as deemed appropriate.

Relevant national professional member organisations and networks (eg, Paediatric Intensive Care Audit Network (PICANet), regional Neonatal Operational Delivery Networks, Children’s Cancer and Leukaemia Group (CCLG), Teenager and Young Adult Cancer (TYAC)) distributed an email invitation to its members on behalf of the study team. A link to the survey was included in the email with the study information sheet attached. Up to four email reminders were used to support response rate. The survey was also publicised on social media and was open May to October 2021.

### Questionnaire

The content of the questionnaire (see box 1) was informed by (i) existing evidence on factors associated with end of life outcomes and parent experience^13^,^18^, (ii) UK clinical guidance^6 19^, and (iii) views of the project’s Parent Advisory Group. Setting-specific versions (NNU; PICU; CYP PTC; TYA PTC) were created. It comprised 54 questions, the majority of which were fixed-response. Final draft versions were piloted with doctors based in the target settings with cognitive interview techniques used to examine question and response form clarity and unambiguity, comprehensiveness and acceptability. Online supplemental material 1 presents the PICU version of the questionnaire. While the survey took place during the COVID-19 pandemic, respondents were instructed to answer questions with respect to their usual (pre-COVID-19) service offer and practices. The survey was hosted on the Qualtrics survey 7 platform.^20^

Box 1 **Questionnaire sections**

- Type of hospital: for example, district general vs tertiary centre.
- Unit characteristics: for example, number of beds, annual ‘caseload’, number of deaths, access to outreach services.
- Unit layout/facilities: for example, facilities for parents.
- Multidisciplinary team: for example, professions represented, keyworker/family liaison role.
- Access to and involvement of palliative care specialists: for example, specialist palliative care teams within the trust/hospital, clinicians within multidisciplinary team (MDT) with palliative care expertise or interest.
- Practices around advance care and end of life planning: for example, protocol for triggering end of life planning, recording and sharing of plans.
- Access to and use of community services: for example, nurse or consultant-led community services, children’s hospices.
- Bereavement support: for example, presence of bereavement specialists in MDT, time with the body, offer of debrief.
- Personal views regarding unit’s end of life care offer: invited to state up to three things doing particularly well and/or up to three things would like to improve.

### Data preparation

Responses were qualitatively checked for missing data. Those with less than 40% visualisation and/or missing data on all/almost all variables (90% or more) were treated as a non-response. Where a unit submitted more than one survey (n=12: 6 NNU, 6 PTC), responses were compared and the more complete response used. Free text responses were categorised by one member of the research team with that categorisation checked by at least two other members of the research team.

### Deriving indicators of the core elements of end of life care

Survey questions capturing information relevant to a distinct characteristic or feature of end of life care service organisation or delivery (ie, the core elements^9^ of models of end of life care) were grouped together. 10 distinct ‘core elements’ were identified which were further organised under three higher-level conceptual domains, see table 1. Each core element was captured by at least one indicator derived from a survey question, or a combination of two or more questions, see table 1. Deriving the indicators was an iterative process informed by existing literature^13^,^18^ and clinical guidelines,^6^ scrutiny of an initial descriptive analysis of the survey data, and two rounds of multistakeholder consultation. Stakeholders included parents (n=13) and representatives (n=68) of key professional groups (eg, medicine, nursing, social work) and specialisms/setting (eg, neonatology, paediatric intensive care, haematology/oncology, palliative care, community nursing, children’s hospices). This ensured the final set of ‘core elements’^9^ (and the wording used to describe them) were meaningful, at the appropriate level of specificity, and applicable across NNUs, PICUs and PTCs.

**Table 1:** Domains and core elements of end of life care and survey-derived indicators

Core element	Indicator	Question(s) used to derive indicator and indicator categories
**CARE OF CHILD AND MANAGEMENT OF CONDITION**		
**Breadth of professions represented in multidisciplinary team (MDT**)	Number of professions represented in MDT	**Many**: (2 or more additional clinical* professions) AND (2 or more non-clinical† professions)**Some**: (2 or more additional clinical professions) AND (1 non-clinical professions)**Few**: (No or 1 additional clinical profession) AND (no non-clinical professions)
**Embeddedness of palliative care expertise in the unit**	Embeddedness of medical palliative care expertise	**Strong**: (Lead doctor for palliative care OR specialist interest/qualified doctor(s)) AND (at least one has protected time in that role)**Partial**: (Lead doctor for palliative care OR specialist interest/qualified doctor(s)) AND (none has protected time)**None**: (No lead doctor for palliative care NOR specialist interest/qualified doctor(s))
	Embeddedness of nursing palliative care expertise	**Strong:** (Lead nurse for palliative care OR specialist interest/qualified nurse(s)) AND (at least one has protected time in that role)**Partial:** (Lead nurse for palliative care OR specialist interest/qualified nurse(s)) AND (none has protected time)**None**: (No lead nurse for palliative care OR specialist interest/qualified nurse(s))
	Involvement of age-appropriate consultant-led palliative care (PC) team	**Strong**: Hospital has (age-appropriate PC team) AND (team regularly attends unit MDT meetings OR ward rounds)**Partial**: Hospital has (age-appropriate PC team) AND (only attend MDT meetings OR ward rounds when invited)**None**: Hospital (does not have age-appropriate PC team) OR (has age-appropriate PC team but never attends MDT meetings OR ward rounds)
**Systems supporting continuity of care**	Recording of advance care plans (ACP) or end of life (EoL) plans	**Yes**: Unit uses at least one type of standardised‡ advance care planning/EoL proforma**No**: Unit does not use at least one type of standardised proforma
**Access and referral to community services which support choice regarding place of care and/or death**	Access to outreach team	**Yes**: (Own outreach team) OR (access to ‘hospital-wide’ outreach team)**No**: Unit does not have access to outreach team
	Refer to doctor-led community service(s)§	**Yes**: Refers to at least one type of doctor-led community service in some or all localities it discharges to**No**: (Does not refer to doctor-led community services in any localities) OR (Does not have such services available in any locality)
	Refer to community nursing¶ or hospice service	**Yes**: Unit refers to at least one type of nurse-led community service in some or all localities unit discharges to**No**: (Does not refer to nurse-led community services in any localities) OR (does not have such services available in any locality)
**CARE OF THE PARENT**		
**Range of parent support available from MDT**	MDT includes professions specialist in psychosocial and spiritual care**	**All**: MDT includes all professions specialist in parent support**Some**: MDT includes 1–2 professions specialist in parent specialist**None:** No parent support specialists on MDT
	Unit has keyworker/ family liaison role	**Yes**: Keyworker/family liaison role in operation**No**: No keyworker/family liaison role in operation
**Availability of on-ward facilities for parents’ physical needs**	On-ward facilities	**All:** Unit has all the following: dedicated toilet, washing and sleeping facilities for parents**Some:** Unit has at least one of the above**None:** None of the above facilities available
**Access to privacy for families**	Availability of side rooms	**Yes**: Ward layout includes at least some single room/cubicles**No**: Ward is open bay
	Availability of dedicated end of life (EoL) space	**Yes**: Dedicated EoL space available on the ward or elsewhere in hospital**No**: No dedicated EoL space available
**BEREAVEMENT CARE**		
**MDT includes staff specialist in bereavement care**	Bereavement care expertise in MDT	**Strong**: Unit has (bereavement lead OR staff specialist trained in bereavement care) AND (staff have protected time for bereavement care)**Partial**: Unit has (bereavement lead OR staff specialist trained in bereavement care) AND (no protected time for bereavement care)**None**: Unit does not have (bereavement lead OR staff specialist trained in bereavement care)
**Immediate bereavement support offer**	Availability of dedicated bereavement suite	**Yes**: Unit has a dedicated bereavement suite/facility**No**: Unit does not have dedicated bereavement suite/facility
	Opportunity for extended time after death	**Yes**: Unit has (own cooling facilities††) OR (refers to children’s hospice cooling facilities)**No**: Unit does not have (own cooling facilities) OR (does not refer to children’s hospice cooling facilities)
	Opportunity for de-brief appointment	**Yes**: De-brief appointment routinely offered to parents**No**: De-brief appointment not routinely offered to parents
**Supporting access to on-going bereavement care**	Routinely refer to on-going bereavement care‡‡	**Yes**: Routinely refers to bereavement care/support**No**: Does not routinely refer to unit bereavement care/support

* Additional clinical: pharmacy, occupational therapy, physiotherapy, dietetics.

† Non-clinical: social work, psychology, play spec/youth worker, chaplaincy/spiritual care.

‡ Standardised EoL/ACP documents: Children and Young People’s Advance Care Plan; ReSPECT form; Limitation of Treatment Agreement.

§ Doctor-led community services: community paediatrician, community paediatrics team, consultant-led paediatric PC team and/or GP-led PC team.

¶ Children’s community nursing services: children’s community nursing team; or nurse-led community paediatric palliative care team (non-hospice)

** Psychosocial-spiritual parent support specialists: social work, chaplaincy, clinical psychology.

†† Cooling facilities: body cooling equipment which allows parents extended time with baby/child after death by delaying when need to be transferred to mortuary or funeral directors (eg, ‘Cuddle Cot’, cooling blanket; cooled bedroom).

‡‡ On-going bereavement care: provided either by: unit’s bereavement worker/team; hospital’s bereavement team (excludes services which only register deaths; and/or other parent-specific bereavement support service).

### Data analysis

Bivariate descriptive statistics (cross-tabulation) and heatmaps were used to explore and present how units represented in the survey ‘scored’ on each indicator.

Following this, we used latent class analysis to identify clusters of similar cases or ‘models of care’ in relation to the core elements of end of life care. Models were performed using the *poLCA* software package^21^ and we tested models of up to 10 classes. Each model was tested 10 times in order to identify the model that globally maximised the log-likelihood function.^21^ Models with negative df were disregarded. Final model selection considered BIC values^22^ and class distributions that were meaningful for the purposes of the analysis. To compare indicators in relation to the groups generated from the latent class analysis, we used Fisher’s exact test. Analyses considered a minimum significance level of 5% and were performed using R V.4.2.1.^23 24^

### Ethical approval

The study was approved by the Department of Health Sciences Research Governance Committee, University of York (Ref: HSRGC/2020/418/G).

## Results

A total of 103 surveys were submitted. Of these, 12 were second respondents from the same unit as another respondent. Removal of these duplicates (see Data preparation section above) left 91 surveys being taken forward for analysis. This represented an overall response rate of 37% though this varied by setting (NNU: n=52, response rate (RR)=29%; PICU: n=19, RR=68%; PTC: n=20, RR=58%). Of the NNUs, 11 were SCBUs, 21 were LNUs and 19 NICUs. 13 PTCs were CYP treatment centres, and 7 TYA centres. Around two thirds of respondents were the unit’s clinical lead/director (n=32/91) or palliative care lead (n=29/91). The remainder (29/91) held other roles (eg, nurse consultant).

### Initial mapping differences in service delivery and practice

**Figure 1:**
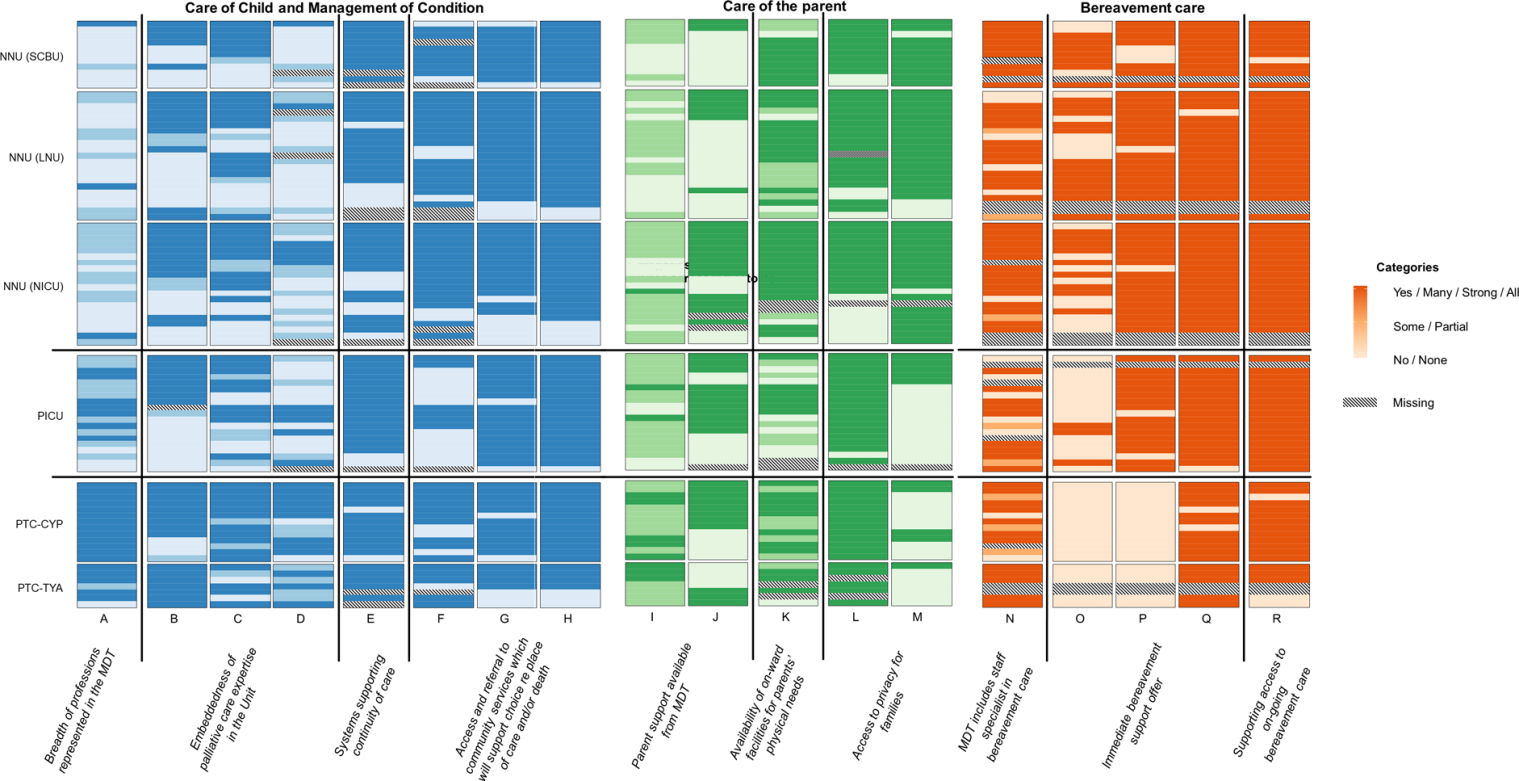
Graphical representation of differences between respondents in terms of core elements of end of life care.

Heatmaps were used to generate graphical representations of differences in the core elements of end of life care service delivery and practice both within each setting (or type of unit represented in the survey) and between the different unit types, see figure 1. This representation conveys differences in end of life care within and between settings. Subsequent sections report findings from the descriptive analyses used to explore these differences for the three domains of end of life care: care of child and management of condition, care of parent during end of life and bereavement care.

### Exploring differences in the care of child and management of condition

Four core elements of service organisation and delivery relevant to the care of the child and management of the condition were captured by the survey (see table 1), namely:

- breadth of professionals represented in the multidisciplinary team (MDT) (one indicator),
- embeddedness of palliative care expertise within the unit (three indicators),
- systems in place to support continuity of care (one indicator),
- access and referral to community services supporting choice over place of care and/or death (three indicators).

PTCs were most likely to report the greatest range of different clinical and non-clinical professions, see table 2. NNUs were most likely to report their MDT did not include any non-clinical professions and just one clinical profession in addition to nursing and medicine. In terms of the extent to which palliative care expertise was embedded in a unit, around half of the MDTs had medical and nursing staff with specialist palliative care expertise or interest. Some or high levels of involvement by a separate age-appropriate palliative care team were reported by almost all PTCs (18/20) but by less than half NNUs (22/48) and PICUs (8/18).

With respect to systems in place to support continuity of care and advance care or end-of-life planning, standardised documentation was typically used (eg, Children and Young People’s Advance Care Plan, Recommended Summary Plan for Emergency Care and Treatment (ReSPECT)). However, such documentation was least likely to be used by NNUs. Finally, in terms of being able to support choice around place of care and/or death, the majority of units across all settings referred to community services. However, PICUs were less likely to report having their own or access to a hospital-based outreach team compared with NNUs and PTCs.

**Table 2:** Core elements of the care of the child/management of the condition by setting

Core element**Indicator**	NNU(n=52)	PICU(n=19)	PTC(n=20)	All(n=91)
**Breadth of professions represented in MDT**				
Number of professions represented in MDT				
Many*	3 (5.8%)	7 (36.8%)	18 (90.0%)	28 (30.8%)
Some	19 (36.5%)	9 (47.4%)	1 (5.0%)	29 (31.9%)
Few	30 (57.7%)	3 (15.8%)	1 (5.0%)	34 (37.4%)
**Embeddedness of palliative care expertise in the unit**				
Embeddedness of medical palliative care expertise				
Strong	26 (50.0%)	8 (44.4%)	16 (80.0%)	50 (55.6%)
Partial	4 (7.7%)	1 (5.6%)	1 (5.0%)	6 (6.7%)
None	22 (42.3%)	9 (50.0%)	3 (15.0%)	34 (37.8%)
Missing†	*0*	*1*	*0*	*1*
Embeddedness of nursing palliative care expertise				
Strong	28 (53.8%)	9 (47.4%)	15 (75.0%)	52 (57.1%)
Partial	6 (11.5%)	4 (21.1%)	3 (15.0%)	13 (14.3%)
None	18 (34.6%)	6 (31.6%)	2 (10.0%)	26 (28.6%)
Involvement of age-appropriate consultant-led palliative care team				
Strong	6 (12.5%)	5 (27.8%)	12 (60.0%)	23 (26.7%)
Partial	16 (33.3%)	3 (16.7%)	6 (30.0%)	25 (29.1%)
None	26 (54.2%)	10 (55.6%)	2 (10.0%)	38 (44.2%)
Missing	*4*	*1*	*0*	*5*
**Systems supporting continuity of care**				
Recording of advance care or end of life plans				
Yes	36 (76.6%)	16 (88.9%)	16 (88.9%)	68 (81.9%)
No	11 (23.4%)	2 (11.1%)	2 (11.1%)	15 (18.1%)
Missing†	*5*	*1*	*2*	*8*
**Access and referral to community services which will support choice regarding place of care and/or death**				
Access to outreach team				
Yes	39 (84.8%)	6 (33.3%)	15 (78.9%)	60 (72.3%)
No	7 (15.2%)	12 (66.7%)	4 (21.1%)	23 (27.7%)
Missing†	*6*	*1*	*1*	*8*
Refer to doctor-led community service(s)				
Yes	41 (78.8%)	17 (89.5%)	15 (75.0%)	73 (80.2%)
No	11 (21.2%)	2 (10.5%)	5 (25.0%)	18 (19.8%)
Refer to community nursing or hospice service				
Yes	45 (86.5%)	18 (94.7%)	17 (85.0%)	80 (87.9%)
No	7 (13.5%)	1 (5.3%)	3 (15.0%)	11 (12.1%)

* sSee table 1 for category definitions.

† Missing reported only if present.

MDTmultidisciplinary teamNNUneonatal unitPICUpaediatric intensive care unitPTCprincipal treatment centre

### Exploring differences in the care of parents

Three core elements of care of parents were captured by the survey (see table 1), namely:

- range of parent support available from the MDT (two indicators),
- availability of on-ward facilities to meet parents’ physical needs (one indicator),
- access to privacy for the family (two indicators).

Compared with NNUs and PICUs, PTC MDTs were more likely to include three professions specialist in different aspects of care of parents with a child at end of life (ie, social work, chaplaincy, clinical psychology), though this was the case for less than half of the PTCs represented, see table 3. Overall, less than half of the units had staff occupying a keyworker or family liaison role (43/91), with this role reported most frequently by PICUs (11/18). In terms of meeting parents’ physical needs, most units (52/85) provided parent-dedicated toilet and washing facilities, as well as sleeping/overnight facilities. However, a third of PICUs (6/17) did not offer these. Finally, most units (73/91) had the potential to offer families privacy because ward(s) included side rooms. However, most PICUs and PTCs did not have access to dedicated, separate end of life spaces. In contrast, the large majority of NNUs (46/51) had such spaces.

**Table 3:** Core elements of the care of parents by setting

Core element**Indicator**	NNU(n=52)	PICU(n=19)	PTC(n=20)	Total(n=91)
**Range of parent support available from MDT**				
MDT includes professions specialist in psychosocial and spiritual care				
All	1 (1.9%)	2 (10.5%)	8 (40.0%)	11 (12.1%)
Some	31 (59.6%)	13 (68.4%)	12 (60.0%)	56 (61.5%)
None	20 (38.5%)	4 (21.1%)	0 (0.0%)	24 (26.4%)
Unit has keyworker/ family liaison role				
Yes	21 (42.0%)	11 (61.1%)	11 (55.0%)	43 (48.9%)
No	29 (58.0%)	7 (38.9%)	9 (45.0%)	45 (51.1%)
Missing*	*2*	*1*	*0*	*3*
**Availability of on-ward facilities for parents’ physical needs**				
On-ward facilities				
All	35 (70.0%)	6 (35.3%)	11 (61.1%)	52 (61.2%)
Some	10 (20.0%)	5 (29.4%)	6 (33.3%)	21 (24.7%)
None	5 (10.0%)	6 (35.3%)	1 (5.6%)	12 (14.1%)
Missing*	*2*	*2*	*2*	*6*
**Access to privacy for families**				
Availability of side rooms				
Yes	38 (76.0%)	17 (94.4%)	18 (100.0%)	73 (84.9%)
No	12 (24.0%)	1 (5.6%)	0 (0.0%)	13 (15.1%)
Missing	*2*	*1*	*2*	*5*
Availability of dedicated EoL space				
Yes	46 (90.2%)	5 (27.8%)	5 (25.0%)	56 (62.9%)
No	5 (9.8%)	13 (72.2%)	15 (75.0%)	33 (37.1%)
Missing	*1*	*1*	*0*	*2*

* Missing is reported only if present.

EoL, end of lifeMDT, multidisciplinary teamNNUneonatal unitPICUpaediatric intensive care unitPTCprincipal treatment centre

### Exploring differences in bereavement care

Three core elements of bereavement care were captured by the survey (see table 1), namely:

- the MDT includes staff specialist in bereavement care (one indicator),
- the immediate bereavement care offer (three indicators),
- supporting access to on-going bereavement care (one indicator).

Most unit MDTs (65/78) included staff specialist trained in bereavement care, though these staff did not always have protected time in that role, see table 4. PICUs were the setting with the greatest proportion of respondents (5/16) reporting none of its staff had specialist training in bereavement care. None of the PTCs had a dedicated bereavement suite nor did most of the PICUs. In contrast, almost two thirds of NNUs had this facility. Almost all NNUs and PICUs reported they were able to offer parents extended time with their child after death through the use of cooling facilities, either on the ward or through referral to a children’s hospice. None of the PTCs reported offering this to parents. Almost all units offered parents de-brief appointments and the majority (79/83) said they routinely referred parents to bereavement support services.

**Table 4:** Core elements of bereavement care by setting

Core element**Indicator**	NNU (n=52)	PICU (n=19)	PTC (n=20)	Total (n=91)
**MDT includes staff specialist in bereavement care**				
Bereavement care expertise in MDT				
Strong	36 (80.0%)	9 (56.2%)	12 (70.6%)	57 (73.1%)
Partial	3 (6.7%)	2 (12.5%)	3 (17.6%)	8 (10.3%)
None	6 (13.3%)	5 (31.2%)	2 (11.8%)	13 (16.7%)
Missing	*7*	*3*	*3*	*13*
**Immediate bereavement support offer**				
Availability of dedicated bereavement suite				
Yes	29 (61.7%)	3 (16.7%)	0 (0.0%)	32 (38.6%)
No	18 (38.3%)	15 (83.3%)	18 (100.0%)	51 (61.4%)
Missing	*5*	*1*	*2*	*8*
Opportunity for extended time after death				
Yes	42 (89.4%)	16 (88.9%)	0 (0.0%)	58 (69.9%)
No	5 (10.6%)	2 (11.1%)	18 (100.0%)	25 (30.1%)
Missing	*5*	*1*	*2*	*8*
Opportunity for de-brief appointment				
Yes	46 (97.9%)	17 (94.4%)	16 (88.9%)	79 (95.2%)
No	1 (2.1%)	1 (5.6%)	2 (11.1%)	4 (4.8%)
Missing	*5*	*1*	*2*	*8*
**Supporting access to on-going bereavement care**				
Routinely refer to on-going bereavement care				
Yes	46 (97.9%)	18 (100.0%)	15 (83.3%)	79 (95.2%)
No	1 (2.1%)	0 (0.0%)	3 (16.7%)	4 (4.8%)
Missing	*5*	*1*	*2*	*8*

MDTmultidisciplinary teamNNUneonatal unitPICUpaediatric intensive care unitPTCprincipal treatment centre

### Testing for distinct alternative models of end of life care

Latent class analysis supported a two-class model (see online supplemental table 1). Class 1 comprised almost all NNUs and most PICUs (15/19). All PTCs, and the remaining PICUs (4/19), were in Class 2. PICUs in Class 1 and Class 2 did not differ in terms of size, location or whether or not the trust also had an NNU or PTC.

Table 5 summarises differences found between Class 1 and Class 2 units with respect to how they profiled on indicators of core elements of end of life care (see online supplemental table 2 for full analytical output). With respect to the domain ‘Care of the child and management of the condition’, Class 1 and Class 2 units differed with respect to two of the four core elements: breadth of professions represented in the MDT (greater breadth in Class 2 units) and embeddedness of palliative care expertise in the unit (stronger involvement of an age-appropriate consultant-led palliative care team in Class 2 units).

With respect to the domain ‘Care of parents’, Class 1 and Class 2 units differed on two of the three core elements: the number of different professions in the MDT specialist in parent support (more holistic parent support offer in Class 2 units), and access to privacy, specifically the availability of dedicated end of life space (more likely in Class 1 units).

Finally, with respect to the domain ‘Bereavement care’, Class 1 and Class 2 units differed in terms of the immediate bereavement support offer. This difference was located in two of the three immediate bereavement support indicators with Class 1 units more likely to have a dedicated bereavement suite(s) and provide parents with the opportunity for extended time with their child after death.

**Table 5:** Differences in end of life care between class 1 and class 2

Domain and core elements	No. indicators where difference found between classes	Indicator(s) differing between classes	Class 1*	Class 2†
**Care of child and condition management**				
Breadth of professions represented in MDT	1/1	Number of additional professions in MDT, including non-clinical	Fewer	Greater
Embeddedness of palliative care expertise in the unit	1/3	Involvement of age-appropriate consultant-led palliative care team	Weaker	Stronger
Systems supporting continuity of care	0/1	*----*	--	--
Access to and referral to community services which support choice regarding place of care and/or death	0/3	*----*	**--**	**--**
**Care of parent(s**)				
Range of parent support available from MDT	1/2	MDT includes professions specialist in psychosocial and spiritual care	Less holistic	More holistic
Availability of on-ward facilities for parents’ physical needs	0/1	----	--	--
Access to privacy	1/2	Availability of dedicated end of life space	More likely	LessLikely
**Bereavement care**				
MDT includes staff specialist in bereavement care	0/1	*----*	--	--
Immediate bereavement support offer	2/3	Availability of dedicated bereavement suite	More likely	LessLikely
		Opportunity for extended time with child after death	More likely	LessLikely
Supporting access to on-going bereavement care	0/1	*----*	--	--

* Class 1 comprised almost all NNUs and most PICUs (15/19).

† Class 2 comprised all PTCs and the remaining PICUs (4/19).

MDTmultidisciplinary team

## Discussion

The primary objective of the survey reported in this paper was, for the first time, to map similarities and differences in the organisation and delivery of care by health services in the UK most likely to be involved in end of life care of babies, children and young people with life-threatening or life-shortening conditions. Further, it sought to identify whether different approaches, or models, of end of life care could be identified. Survey findings are foundational to subsequent stages of the ENHANCE study which is seeking to increase our understanding of the aspects of service organisation and delivery at end of life that impact children’s and parents’ outcomes and experiences.

The survey was concerned with three domains of care: care of the child and management of their condition, care of the parent during end of life, and bereavement care. Within each domain, the core elements of care likely to impact child/parent outcomes and experience were specified based on existing evidence and clinical guidance, with indicators of the core components generated from the data collected by the survey (table 1). As such, this core elements/indicators framework has a potential application as an audit/service review tool as well as for future research. Importantly, our findings indicate variability in aspects of service provision and delivery that matter when a baby or child is at end of life.

To summarise, with respect to the ‘care of the child and management of the condition’ domain, PTCs were most likely to have the widest range of clinical and non-clinical professions represented on the MDT, and for the MDT to include medical and nursing staff with palliative care expertise. They were also more likely to have access to the specialist palliative care service based in their hospital and an outreach team, and thus, able to support greater choice on place of care and/or death.

In terms of the ‘care of the parent’ domain, compared to PTCs, relatively few MDTs in NNUs and PICUs had professions specialist in psychosocial and spiritual care. PICUs were also least likely to have comprehensive on-ward personal care/sleeping facilities for parents. The lack of dedicated end of life spaces for families in PICUs (where ~15% of child deaths occur) and PTCs contrasts strongly with their near universal availability in NNUs. Recent work has raised awareness of this aspect of end of life care in paediatric settings^25^,^27^ highlighting the importance of paying attention to the physical environment when planning and delivering end of life care.^28^,^30^

Our final domain of end of life care was ‘bereavement care’. Again, we found differences between settings. NNUs emerged as most likely to be providing bereavement care in multiple ways and to have staff specialist in bereavement care with protected time for this role. None of the PTCs were routinely offering parents the opportunity to delay the transfer of their child’s body to the mortuary or funeral directors through the use of cooling facilities at a local hospice or at home (eg, cooling blankets/cots) or, for a shorter duration, on the ward. This runs counter to evidence on the value parents place on the opportunity for extended time with their child after death and how this can positively impact on the grieving process and bereavement outcomes.^18^

As well as revealing differences in end of life care provision and practices between settings, we also found a widespread absence of family liaison/keyworker roles across all settings. It is possible that financial constraints (and/or reduction in the incomes of charities who commonly fund these roles) have led to a reduction in the number of units able to incorporate this role into the MDT. However, evaluations of this role reveal the multiple ways it supports parents (and the wider family), including emotional support, advocacy, service navigation and providing or enabling access to practical/financial support.^31^ Taken alongside evidence on parents’ needs,^14 25 27^ the critical and irreplaceable role parents play in the care and support of their child at end of life^31^and quality of life outcomes, regarding parent support roles as non-critical is, we would argue, mis-guided.

Finally, we investigated whether these core elements of end of life care consistently clustered, or co-occurred, together thereby revealing distinct, alternative models of end of life care. Two models were identified. However, the models were not found across all settings suggesting instead broad overall differences in what and how end of life care is provided by PTCs compared to PICUs and NNUs. There are likely to be a number of explanations for this including the needs and characteristics of the patient group and differences in terms of place of death (on unit v home/hospice).^32^

### Study limitations

Response rates were lower than hoped, particularly for non-intensive neonatal settings. The resultant sample size means the latent class analysis^33^ should be treated as exploratory. Data was collected from a sole respondent with the expectation that they would be able to reliably report on multiple aspects of service organisation, delivery and practice: this may not be the case. Despite careful piloting, it is possible that respondents’ understanding of terms or phrases used to described particular aspects of end of life care (eg, referral to bereavement support services, de-brief appointment) varied.

## Conclusion

Survey findings suggest UK settings most likely to be involved in the care of babies, children and young people at end of life differ in the care and support provided or offered. Crucially, these differences are located in areas of service organisation, delivery and practice which existing evidence indicates matter to families, and impact patient/parent outcomes and experiences.

The reasons for these differences are likely to be multiple. The wider hospital context (eg, availability of hospital-wide services relevant to end of life care), community context (eg, access to children’s hospice services), and the funding allocated or available will necessarily constrain how NNUs, PICUs and PTCs provide end of life care. Thus, the findings are relevant to those in strategic positions within NHS trusts as well as services themselves.

Subsequent stages of the ENHANCE study will generate evidence on the relative importance and contribution of the core elements of end of life care investigated by this survey to the outcomes and experiences of children and their parents, and the possible benefits of additional funding in terms of patient outcomes. Finally, replicating this study in the UK to achieve a higher response rate is recommended. Furthermore, the indicators of end of life care captured by the survey are relevant across a range of healthcare settings and systems, meaning that core sections of the survey would be amenable for use by researchers and healthcare providers in other countries.

## supplementary material

10.1136/spcare-2023-004673 online supplemental table 1

10.1136/spcare-2023-004673 online supplemental file 1

## Data Availability

Data are available upon reasonable request.
