# Supplementary material for: End of life care in paediatric settings: UK national survey
Source: BMJ Support Palliat Care. 2024 Nov 28;15(2):e004673. doi: 10.1136/spcare-2023-004673 (PMC11874335; doi:10.1136/spcare-2023-004673)
Supplement: online supplemental table 1 [file spcare-15-2-s001.pdf]

Supplementary Table 1: Model fit evaluation information

	log-likelihood	Residual df	BIC	aBIC	cAIC	likelihood-ratio	Entropy
2 Classes	-956.225	40	2142.503	1981.531	2193.503	821.5121	0.962
3 Classes	-915.817	14	2178.97	1935.934	2255.97	766.8837	0.897
4 Classes	-886.579	-12	2237.776	1912.676	2340.776	726.3195	0.922
5 Classes	-871.258	-38	2324.416	1917.252	2453.416	689.9039	0.902

Supplementary Table 2: Cluster/class response percentages within variables

	Class 1 (N=65)	Class 2 (N=26)	Total (N=91)	p value
**Unit/centre Type**				<0.001[1]
NNU	50 (76.9%)	2 (7.7%)	52 (57.1%)	
PICU	15 (23.1%)	4 (15.4%)	19 (20.9%)	
PTC	0 (0.0%)	20 (76.9%)	20 (22.0%)	
**Care of child & condition management**				
Breadth of professions represented in MDT				
No. professions represented in Unit/Centre's MDT				<0.001[1]
Few	33 (50.8%)	1 (3.8%)	34 (37.4%)	
Some	28 (43.1%)	1 (3.8%)	29 (31.9%)	
Many	4 (6.2%)	24 (92.3%)	28 (30.8%)	
Embeddedness of palliative care expertise in the Unit				
Embeddedness of medical palliative care expertise in Unit/Centre's MDT				0.024[1]
None	30 (46.2%)	4 (16.0%)	34 (37.8%)	
Partial	4 (6.2%)	2 (8.0%)	6 (6.7%)	
Strong	31 (47.7%)	19 (76.0%)	50 (55.6%)	
missing	*0*	*1*	*1*	
Embeddedness of nursing palliative care expertise in Unit/Centre's MDT				0.053[1]
None	23 (35.4%)	3 (11.5%)	26 (28.6%)	
Partial	9 (13.8%)	4 (15.4%)	13 (14.3%)	
Strong	33 (50.8%)	19 (73.1%)	52 (57.1%)	
Involvement of age-appropriate consultant-led palliative care team				<0.001[1]
None	34 (56.7%)	4 (15.4%)	38 (44.2%)	
Partial	18 (30.0%)	7 (26.9%)	25 (29.1%)	
Strong	8 (13.3%)	15 (57.7%)	23 (26.7%)	
missing	*5*	*0*	*5*	
Systems supporting continuity of care				
Recording of advance care or end of life plans				0.536[1]
No	12 (20.3%)	3 (12.5%)	15 (18.1%)	
Yes	47 (79.7%)	21 (87.5%)	68 (81.9%)	
missing	*6*	*2*	*8*	
Access and referral to community services which support choice re place of care and/or death				
Access to outreach team				1.000[1]
No	16 (27.6%)	7 (28.0%)	23 (27.7%)	
Yes	42 (72.4%)	18 (72.0%)	60 (72.3%)	
Missing	*7*	*1*	*8*	
Refer to doctor-led community service(s)				0.382[1]
No	11 (16.9%)	7 (26.9%)	18 (19.8%)	
Yes	54 (83.1%)	19 (73.1%)	73 (80.2%)	
Refer to community nursing or hospice service(s)				0.723[1]
No	7 (10.8%)	4 (15.4%)	11 (12.1%)	
Yes	58 (89.2%)	22 (84.6%)	80 (87.9%)	
**Care of parent(s)**				
Parent support available from MDT				
**Care of parent(s)**				
Presence of professions on MDT specialist in welfare/family support, emotional & spiritual care				<0.001[1]

None	24 (36.9%)	0 (0.0%)	24 (26.4%)	
Some	41 (63.1%)	15 (57.7%)	56 (61.5%)	
All	0 (0.0%)	11 (42.3%)	11 (12.1%)	
Presence of staff on MDT with keyworker or family liaison role				0.352[1]
No	34 (54.8%)	11 (42.3%)	45 (51.1%)	
Yes	28 (45.2%)	15 (57.7%)	43 (48.9%)	
missing	*3*	*0*	*3*	
Availability of on-ward facilities for parents' physical needs				
On-ward facilities for parents				0.693[1]
None	8 (13.1%)	4 (16.7%)	12 (14.1%)	
Some	14 (23.0%)	7 (29.2%)	21 (24.7%)	
All	39 (63.9%)	13 (54.2%)	52 (61.2%)	
missing	*4*	*2*	*6*	
Access to privacy for families				
Availability of side rooms				0.016[1]
No	13 (21.0%)	0 (0.0%)	13 (15.1%)	
Yes	49 (79.0%)	24 (100.0%)	73 (84.9%)	
missing	*3*	*2*	*5*	
Availability of dedicated end of life space				<0.001[1]
No	12 (19.0%)	21 (80.8%)	33 (37.1%)	
Yes	51 (81.0%)	5 (19.2%)	56 (62.9%)	
missing	*2*	*0*	*2*	
**Bereavement care**				
MDT includes staff specialist in bereavement care				
Bereavement care expertise in MDT				0.488[1]
None	11 (19.3%)	2 (9.5%)	13 (16.7%)	
Partial	5 (8.8%)	3 (14.3%)	8 (10.3%)	
Strong	41 (71.9%)	16 (76.2%)	57 (73.1%)	
missing	*8*	*5*	*13*	
Immediate bereavement support offer				
Availability of dedicated bereavement suite				<0.001[1]
No	29 (49.2%)	22 (91.7%)	51 (61.4%)	
Yes	30 (50.8%)	2 (8.3%)	32 (38.6%)	
missing	*6*	*2*	*8*	
Opportunity for extended time after death				<0.001[1]
No	7 (11.9%)	18 (75.0%)	25 (30.1%)	
Yes	52 (88.1%)	6 (25.0%)	58 (69.9%)	
missing	*6*	*2*	*8*	
Opportunity for de-brief appointment				0.071[1]
No	1 (1.7%)	3 (12.5%)	4 (4.8%)	
Yes	58 (98.3%)	21 (87.5%)	79 (95.2%)	
missing	*6*	*2*	*8*	
Supporting access to on-going bereavement care				
Routinely refer to bereavement care/support				0.071[1]
No	1 (1.7%)	3 (12.5%)	4 (4.8%)	
Yes	58 (98.3%)	21 (87.5%)	79 (95.2%)	
missing	*6*	*2*	*8*	

MDT: Multidisciplinary team; EoL: End of Life. Missing is reported only if present.

[1]Fisher's Exact Test
