# Supplementary material for: End of life care in paediatric settings: UK national survey
Source: BMJ Support Palliat Care. 2024 Nov 28;15(2):e004673. doi: 10.1136/spcare-2023-004673 (PMC11874335; doi:10.1136/spcare-2023-004673)
Supplement: online supplemental file 1 [file spcare-15-2-s002.pdf]

Block 1 - Introduction

End of Life Care for Infants, Children and Young People: a mixed methods evaluation of current practice in the UK (ENHANCE)

Stage 1 survey: Paediatric Intensive Care Unit (PICU) leads / directors

This survey concerns how your Unit manages the care of babies who are at end of life.

The ENHANCE study defines "end of life" as:

- where, with respect to a child admitted to your Unit, you would not be surprised to hear, within the next 12 months, that they had died

  AND / OR

- during an admission to your Unit, the decision is made to stop active treatment or withdraw life-sustaining treatment(s).

We recognise this definition of end of life may be broader than survey respondents' usual understanding of this term. The above definition is repeated at relevant sections in the survey.

Completing the survey
It should take no longer than 25 - 30 minutes to complete.
Please note: 'free text' boxes will expand as your type into them.

If you have a palliative care lead in your team, you may want to delegate completing this survey to them. To do this, please forward the invitation email to them.

Two questions ask for annual figures from your Unit's 2019 annual audit/report. If you do not have these figures to hand, please provide estimates.

The deadline for response is *Friday, 30th July 2021*

If you have any questions about the survey or study more generally, please don't hesitate to contact us at dohs-enhance@york.ac.uk

Thank you for your help with this important research.

Please tick the statement which best describes your role.

- ○ Clinical Lead/Director of Unit
- ○ Unit's palliative care lead
- ○ Other. *Please describe:* ______

Have you already read the information sheet for this study?

No	Yes
○	○

End of Life Care for Infants, Children and Young People (ENHANCE)

Study Information Sheet: Workstream 1 (Clinical Leads)

You are being invited to take part in Workstream 1 of the above study. The purpose of this workstream is to map and describe the ways children's palliative and end of life care is currently being provided. Taking part involves completing a short on-line questionnaire. Please read the information below to help you decide whether or not to take part.

What is this research about?
The ENHANCE study is concerned with palliative and end of life care for infants, children and young people. It will compare the current ways this is provided (i.e. delivery models) in terms of the outcomes and experiences of, and costs to families

and the NHS. A first, and foundational, workstream (WS1) will identify models of palliative and end of life care within children's cancer services, and paediatric intensive care and neonatal units across the UK. This will be achieved by conducting a survey of clinical leads and interviewing Chairs of regional paediatric palliative care networks. Subsequent workstreams will investigate and evaluate the different delivery models identified by WS1.

Why is this research important?
Around 4500 children die in England and Wales every year, but services vary in how they manage and respond to palliative care needs and end of life care. There is little evidence on the models of care currently being used, and whether it affects quality of care and patient/family experience and outcomes for children and their families. As a result, recommendations within NICE and other national clinical guidance are based on low quality evidence. This study will provide the evidence needed to help ensure children and families receive the best care and support.

Who is doing the study?
The study is being led by a team based at the University of York. The study is funded by the National Institute for Health Research (NIHR) (Health Services and Delivery Research (HS&DR) Programme) (Study Reference: NIHR129213).

Why am I been asked to participate?
You are being invited to take part in Workstream 1 of this study: the survey of clinical leads. All clinical leads of children's cancer services, paediatric intensive care and neonatal units in the UK are being asked to take part.

Do I have to take part?
No. Taking part is voluntary.

What will be involved if I take part in this study?
We would like you to complete an online survey about the service you lead in terms of its organisational features (e.g. setting, team make-up and skills, populations served) and practices, pathways and partnerships implemented in palliative or end-of-life care situations. This should take you no longer than 20 minutes. At the end of the survey, we also ask if your service might be interested in being involved in the later, evaluative workstreams.

What are the possible risks and benefits of taking part?

You are being invited to take part in this survey in your professional role. It will not be possible for your responses to be anonymised, but you won't be able to be identified through any outputs that are produced as a result of the study. There are no direct benefits to you if you take part in this research study but taking part will contribute to improving the evidence base on palliative and end-of-life care of babies, children and young people.

Can I withdraw from the study at any time?
Once you have submitted the survey, you cannot withdraw from the study. However, it will be possible to request the opportunity to revise your response for up to 14 days after submitting the survey.

How will the information and personal data I give be handled?
All the information we collect will be stored and managed in accordance with the General Data Protection Regulation (GDPR) and the University of York's research data management policy. It will only be used for the purposes of this study. The information you provide will be given a unique reference number and will be stored separately from any personal information that we collect from you during the course of the study. More information is provided through the links below:
https://www.york.ac.uk/records-management/dp/
https://www.york.ac.uk/records-management/dp/guidance/gdprcompliantresearch/
https://www.york.ac.uk/records-management/dp/your-info/generalprivacynotice/
https://www.york.ac.uk/healthsciences/research/trials/trials-gdpr/
https://www.york.ac.uk/healthsciences/research/trials/trials-gdpr/research-partcipants/

What will the information I provide be used for?
We will use the survey data, and other data collected in this workstream, in two main ways. First, it will be analysed to create a typology of the different approaches being taken by services to provide end of life care to babies, children and young people. Second, we will use the data to identify services which could act as exemplars of the different delivery models for the main evaluative elements of the study.

We will also publish workstream findings in an academic/research journal and seek opportunities to present to key national bodies and special interest groups. We will also produce a summary of key findings. You will be asked if you want to receive this at the end of the survey.

You will not be identifiable in any project report or presentation. Similarly, without seeking specific permission, we will not use the name of your service, or information which could identify it, in any written or verbal outputs. We will only use personal information collected by the survey to manage your participation in the study and provide you with a summary of the findings.

Who else might see the information I provide?
The University of York is the sponsor for this study. They are responsible for making sure all the information collected during this study is looked after and used properly. This means that individuals from the University of York and regulatory organisations (e.g. organisations that fund or approve the research) may need access to your research record as part of an audit of data collection and management processes. This means that, outside of the research team, the only people who can access personal/identifiable information will be designated sponsor representatives. No personal/identifiable data would be passed on to those conducting any such audit. In accordance with University of York policy, all research data and records are kept for ten years once a study is complete and is then destroyed.

Who has reviewed and approved this study?
This workstream of the project has been approved by the University of York's Health Sciences Research Governance Committee.

Who do I contact for more information about the study?
We'd be very happy to hear from you if you have any questions about taking part in the study. Please contact the study manager, Andrew Papworth (andrew.papworth@york.ac.uk), or the study lead, Lorna Fraser (lorna.fraser@york.ac.uk).

Who do I contact in the event of a complaint?
If you have concerns about the study or data protection issues, or wish to make a complaint, please contact the University of York Data Protection Officer (Email: dataprotection@york.ac.uk) or the Chair of the Health Sciences Research Governance Committee, Stephen Holland (stephen.holland@york.ac.uk).

If you are still not satisfied, you can contact the University of York's Contracts and Sponsorship Manager, Michael Barber (michael.barber@york.ac.uk)

If you are unhappy with the way your personal data has been handled, you have the right to complain to the University's Data Protection Officer at dataprotection@york.ac.uk; if you are still unsatisfied, you can report concerns to the Information Commissioner's Office at https://www.ico.org.uk/make-a-complaint.

Thank you for taking the time to read this information sheet.

Block 2 - Hospital and Trust/Board

ABOUT YOUR HOSPITAL AND NHS TRUST

This section collects background information about your NHS Trust and the hospital where your Unit is based.

What is the name of the NHS Trust which your hospital is part of?

What is the name of your hospital?

Does your **hospital** have a Lead Nurse for paediatric palliative care?

- No
- Yes
- Don't know

Does your **hospital** have a Lead Consultant for paediatric palliative care?

- No
- Yes
- Don't know

Does your region have a clinical network for paediatric palliative care?

- ○ No
- ○ Yes. If known, please provide name of network lead/chair: [ ]
- ○ Don't know

Block 3 - Unit

ABOUT YOUR UNIT
This section is about your Unit's setting, size and caseload in 2019. Please provide estimates if you do not have figures to hand.

Is there a High Dependency Unit co-located / attached to the PICU?

- ○ No
- ○ Yes. **Please only answer this survey with respect to the PICU**.

What is the maximum capacity of your Unit?

[ ]

Please enter the following data for your Unit for **2019**. If you do not have exact figures to hand, either provide an estimate or tick 'not known / unavailable'

		Not known / unavailable
Total number of admissions	[ ]	☐
Average length of stay (days)	[ ]	☐
Longest stay (days)	[ ]	☐
Average occupancy (%)	[ ]	☐

Please provide the following data on Unit deaths in 2019. If you do not have exact figures to hand, either provide an estimate or tick 'not known / unavailable'.

	Number	Not known / unavailable
Total number of deaths **on the Unit**	[ ]	☐

	Number	Not known / unavailable
Total number of babies **transferred elsewhere** at end stage		☐
Number of transfers to hospice at end stage		☐
Number of transfers to home at end stage		☐

Block 4 Core Team

THE CORE TEAM

This section asks about the make-up of the core team, and whether the Unit has any staff with specialisms/specialist training directly relevant to end of life care.

***To remind you**, this study defines end of life as: where, with respect to a child admitted to your Unit, you would not be surprised to hear, within the next 12 months, that they had died, AND/OR during an admission to your Unit, the decision is made to stop active treatment or withdraw life-sustaining treatment(s).*

In addition to medical and nursing staff, which professions are part of the Unit's core multi-disciplinary team? *(Please include all staff regularly and routinely involved in MDT meetings.)*

- ☐ Pharmacy
- ☐ Occupational therapy
- ☐ Physiotherapy
- ☐ Dietetics
- ☐ Social work
- ☐ Clinical psychology
- ☐ Play specialist
- ☐ Youth work
- ☐ Chaplaincy/spiritual care
- ☐ Other. *Please describe*: ____

Does your Unit have a Lead Consultant for palliative care?

- ○ No

- ○ Yes, always directly involved in all end of life situations
- ○ Yes, may be involved directly or act in advisory/consultancy role
- ○ Other. *Please describe:* [ ]

Does your Unit have a Lead Nurse for palliative care?

- ○ No
- ○ Yes, always directly involved in all end of life situations
- ○ Yes, may be directly involved or act in consultancy role
- ○ Other. *Please describe:* [ ]

Does the team include one or more family liaison nurses*, or an equivalent role?
[*Hover here for definition]

- ○ No
- ○ Yes
- ○ Other. *Please insert details*: [ ]

Are any of your Unit's doctors specialist trained (Level 3 or 4)*, or have a specialist interest in, in paediatric palliative care? (*Tick all which apply.*) **[*Hover here for definition]**

- ☐ No
- ☐ Yes, specialist trained. *Please tell us how many*: [ ]
- ☐ Yes, specialist interest. *Please tell us how many*: [ ]
- ☐ Don't know

Are the programmed activities (PA) of any of these doctors allocated to palliative care?

- ○ No
- ○ Yes. *If known, please tell state total PA/week allocated to palliative care* [ ]

Do any of your Unit's nursing team have specialist training or a specialist interest in paediatric palliative care? (*Tick all which apply.*)

- ☐ No
- ☐ Yes. specialist trained. *Please tell us how many*: ____
- ☐ Yes, specialist interest. *Please tell us how many*: ____
- ☐ Don't know

Do any of these nurses have time specifically allocated to palliative care?

- ○ No
- ○ Yes. *If known, please tell us the total number of hours/week allocated to palliative care*. ____

We would like to collect some additional information about your Unit's funding, resources and expenditure. Please provide contact details for the individual who will hold this information (e.g. operational lead, business manager).

Name: ____

Email: ____

Block 5 Unit Layout and facilities

UNIT LAYOUT AND FACILITIES

This section asks about your Unit's layout and facilities for parents. Where relevant (e.g. facilities for parents), please respond with respect to pre-COVID.

How is your Unit arranged?

- ○ Open bay
- ○ Single rooms/cubicles
- ○ Mix of open bay and cubicles
- ○ Other. *Please describe:* ____

Which of the following parent/family facilities does your Unit have?

- ☐ Dedicated toilets
- ☐ Dedicated bathrooms
- ☐ Dedicated parents' "day room"

Does your Unit have access to a room/suite, designed to be home-like, for use when a child is close to death (that is, final days/hours)?

- ○ No
- ○ Yes, located on the Unit
- ○ Yes, located elsewhere in the hospital. *Please tell us where:* ________

What types of overnight accommodation are available to parents? (*Tick all which are available*)

- ☐ Hospital has generic provision for family members
- ☐ Hospital has dedicated parent accommodation
- ☐ Sleeping facilities within the Unit
- ☐ Other. *Please describe* ________
- ☐ None of the above

Block 6 Working with hospital's palliative care service

WORKING WITH THE HOSPITAL'S SPECIALIST PALLIATIVE CARE SERVICES
This section asks about specialist palliative care services at your hospital and any ways your Unit works with them.

To your knowledge, which of the following consultant-led, specialist palliative care services does your hospital have?

- ☐ Consultant-led **neonatal** palliative care service
- ☐ Consultant-led **paediatric** palliative care service
- ☐ Consultant-led **all-age** palliative care service
- ☐ Consultant-led **adult** palliative care service

☐ **None of the above**

Does your Unit refer to or involve this/these services?

	Yes	No
» Consultant-led **neonatal** palliative care service	○	○
» Consultant-led **paediatric** palliative care service	○	○
» Consultant-led **all-age** palliative care service	○	○
» Consultant-led **adult** palliative care service	○	○
» **None of the above**	○	○

Please tick the service you work with most frequently.

○ » Consultant-led **neonatal** palliative care service

○ » Consultant-led **paediatric** palliative care service

○ » Consultant-led **all-age** palliative care service

○ » Consultant-led **adult** palliative care service

○ » **None of the above**

Please complete the remainder of this section with respect to the specialist palliative care service you work with most frequently.

Please provide name and, if available, contact details for the clinical lead of this service.

Name

Email

Do any of your core team have a joint post with this specialist palliative care service?

○ No

- ○ Yes

Who holds a joint post? *(Tick all that apply)*

- ☐ Doctor
- ☐ Nurse
- ☐ Other. *Please specify:* ______

Tick the statement which best describes this service's availability during weekdays and at weekends.

	Working hours	*24 hours*	*Other*
Weekday availability	○	○	○
Weekend availability	○	○	○

Does this service also work into the community? (e.g. families' homes, local hospices)

- ○ No
- ○ Yes
- ○ Don't know

With respect to children who are at end of life*, for around how many does your Unit involve this specialist palliative care service? ***[*Hover here for definition]***

all	most	some	a few	none
○	○	○	○	○

What scenario(s) **routinely*** trigger referral to this service. *(Tick all that apply.)* ***[*Hover here for definition of routinely]***

- ☐ Pre-existing life-limiting diagnosis
- ☐ Decision required regarding use of life-sustaining treatment(s)
- ☐ Withdrawal of life-sustaining treatments
- ☐ Clinical judgement is death is possible during current admission
- ☐ Clinical judgement is death is inevitable during current admission
- ☐ Clinical judgement is death is possible in ensuing 12 months

- ☐ Transfer home for final days/hours
- ☐ Transfer to hospice for final days/hours
- ☐ Other. *Please describe:* ________

Please describe the ways this service may be involved in the care of a children (and families) on the Unit.

- ☐ Advance care planning
- ☐ Planning for end stage
- ☐ Symptom management
- ☐ Management of transfer to home/hospices for final days/hours
- ☐ Psychological care
- ☐ Liaison with other agencies
- ☐ Other. *Please describe*: ________

Do members of this specialist palliative care service attend your Unit's MDT meetings

- ○ No
- ○ Yes, attend regularly, whether or not involved in a specific case
- ○ Yes, but only when invited
- ○ Other. *Please describe*: ________

Do members of this service attend ward rounds?

- ○ No
- ○ Yes, attend regularly, whether or not involved in a specific case
- ○ Yes, but only when invited
- ○ Other. *Please describe:* ________

Please list below any other palliative care professionals or services based in your hospital which your Unit refers to or works with. Please also tell us the stage at which they become involved, and if they work into the community.

	When does involvement typically start?	Tick if also works into community

	When does involvement typically start?	Tick if also works into community
1. ☐	⌄	☐
2. ☐	⌄	☐
3. ☐	⌄	☐

Block 7 Planning for end of life

PLANNING FOR END OF LIFE

This section concerns planning for end of life. This may occur in parallel with active treatment.

***To remind you**, this study defines end of life as: where, with respect to a child admitted to your Unit, you would not be surprised to hear, within the next 12 months, that they had died, AND / OR during an admission to your Unit, the decision is made to stop active treatment or withdraw life-sustaining treatment(s).*

Which of the following situations trigger your Unit to plan for end of life, or instigate a review of existing plans? *(Tick all which apply)*

- ☐ Child admitted with pre-existing life-limiting diagnosis
- ☐ Decision required regarding use of life-sustaining treatments
- ☐ Withdrawal of life-sustaining treatments
- ☐ Clinical judgement is death is possible during current admission
- ☐ Clinical judgement is death is inevitable during current admission
- ☐ Clinical judgement is death is possible in ensuing 12 months
- ☐ Transfer home for final days/hours
- ☐ Transfer to hospice for final days/hours
- ☐ Other. *Please briefly describe*: ________
- ☐ None of the above

How are plans for end of life recorded by your Unit? *(Tick all which apply)*

- ☐ Children and Young People's Advance Care Plan (CYPACP)
- ☐ ReSPECT form
- ☐ Limitation of Treatment Agreement (LOTA)
- ☐ Proforma created by own Unit
- ☐ Recorded in case notes only
- ☐ Other. *Please describe:* ____

Which people/organisations **routinely*** receive plans for end of life? For each, please tell us how plans are shared. ***[*Hover here for definition of routinely]***

Receive EoL plan?		Paper copy	Email attachment	Shared electronic access
☐	Family	☐	☐	☐
☐	GP	☐	☐	☐
☐	Ambulance Service	☐	☐	☐
☐	Hospice (if involved)	☐	☐	☐
☐	NHS community services (if involved)	☐	☐	☐

Does your Unit have a specific protocol or pathway for managing any of the following? *(Tick all which apply)*

- ☐ Transfer to home for final days/hours
- ☐ Transfer to hospice for final days/hours
- ☐ Planned withdrawal of life-sustaining treatment(s)
- ☐ Decision to withhold life-sustaining treatments(s)
- ☐ Brain death

Block 8 Community Services

COMMUNITY SERVICES

This section concerns community services in the locality, or localities, your Unit serves.

Does your Unit have its own outreach team (i.e. team providing care in families' homes)?

- ○ No
- ○ We don't have our own outreach team, but there is one in the hospital we can refer to. *Please provide brief details*: ________
- ○ We have our own outreach team.

To your knowledge, which of the following community services are available in the locality/localities your Unit serves? (*Please report as per pre-COVID*.)

Please enter a response for each service listed

	All localities	*Some localities*	*Not available*
Children's hospice	○	○	○
Community paediatrician	○	○	○
Community paediatrics team	○	○	○
Children's community nursing team	○	○	○
	All localities	*Some localities*	*Not available*
Consultant-led community paediatric palliative care team (non-hospice)	○	○	○
Nurse-led community paediatric palliative care team (non-hospice)	○	○	○
GP-led paediatric palliative care service	○	○	○

At what stage does your Unit typically refer to these community services?

	Death expected within 12 months	*Death possible within 12 months*	*Determined on case by case basis*	*Do not refer to*
» Children's hospice: inpatient and hospice-at-home	○	○	○	○
» Community paediatrician	○	○	○	○

	Death expected within 12 months	*Death possible within 12 months*	*Determined on case by case basis*	*Do not refer to*
» Community paediatrics team	○	○	○	○
» Children's community nursing team	○	○	○	○
» Consultant-led community paediatric palliative care team (non-hospice)	○	○	○	○
» Nurse-led community paediatric palliative care team (non-hospice)	○	○	○	○
» GP-led paediatric palliative care service	○	○	○	○

Please list below all the children's hospices you refer to.

Block 9 Bereavement support

BEREAVEMENT SUPPORT

This section asks about the different ways units may support bereaved parents.

Does the Unit's team include any staff specialist trained in bereavement support?

- ○ No
- ○ Yes
- ○ Don't know

Does your Unit have someone with lead responsibility for bereavement support?

- ○ No
- ○ Yes
- ○ Don't know

Do any of these staff have time specifically allocated to bereavement support work?

- ○ No
- ○ Yes
- ○ Don't know

From the list below, please tick all which your Unit **routinely*** offers or provides to a family when their child dies on the Unit. ***[*Hover here for definition of 'routinely']***

- ☐ Opportunity for memory/memento-making
- ☐ Dedicated bereavement suite
- ☐ 'Cooling equipment' (e.g cooling blanket/mattress) on the Unit to delay need to transfer child to mortuary
- ☐ 'Cooling equipment' for use at home
- ☐ Referral to children's hospice to use their 'cooling facilities' (e.g. 'cold bedroom')
- ☐ Referral to Unit's bereavement worker/team
- ☐ Referral to other parent-specific bereavement support service.
- ☐ Referral to hospital bereavement team (excludes services which only register deaths)
- ☐ Referral to chaplaincy service (sometimes known as spiritual/pastoral care service)
- ☐ Written information about bereavement support services and charities
- ☐ De-brief appointment with clinical team
- ☐ Inform GP that family has experienced a bereavement
- ☐ Other. *Please briefly describe*: __________

Block 10 Support from charities

SUPPORT FROM CHARITIES

This section briefly asks about the charitable support your Unit receives.

Do any charities specifically fundraise for your Unit?

- ○ No
- ○ Yes. *Please tell us the name of the charity/ies:* __________
- ○ Don't know

To your knowledge, are any posts in your Unit all or part-funded by charity/ies?

- No
- Yes. *Please specify which post(s)* ____
- Don't know

To your knowledge, in the past five years, have any of your Unit's capital building projects been all/partly funded by charitable donation?

- No
- Yes. *Please specify building project:* ____
- No capital building projects in past five years
- Don't know

Does your Unit have particular roles/activities which are undertaken by volunteers? (*Please respond with respect to pre-COVID.*)

- No
- Yes. *Please describe:* ____
- Don't know

Block 11 Summing up

SUMMING UP
In this final section we ask for brief personal reflections on care at end of life care provided by your Unit.
To remind you*, the study's definition of end of life is: where, with respect to a child admitted to your Unit, you would not be surprised to hear, within the next 12 months, that they had died AND/OR during an admission to your Unit, the decision is made to stop active treatment or withdraw life-sustaining treatment(s).*

Please briefly describe **up to** three things your Unit does particularly well in the care of children at end of life.

1.
2.
3

Please briefly describe **up to** three things you would like to improve in the way your Unit cares for children at end of life. This could include things about your own service/practices *and/or* the availability/practices of other services.

1.
2.
3.

Block 12 Summing up 2

Finally, what do you think about defining end of life so it includes patients whose "death within the next 12 months would not surprise you"?

- ○ Meaningful and relevant to the patient group I care for
- ○ Not meaningful or relevant to the patient group I care for

If you would like to add a comment about this, please use the space below.

Before submitting your survey, please tell us how would you like to stay in touch / continue involvement with the ENHANCE study. *(Tick all which apply.)*

- ☐ Please send me survey findings, and findings from later stages
- ☐ I am interested in being contacted about the possibility of my team being involved in **Stage 2** which is investigating the experiences of parents and clinical teams. (Ticking this box does not commit you to getting involved.) *[Sites involved in Stage 2 will: i) help us to identify bereaved parents and provide them with study information; and ii) host a focus group for staff.]*

Please provide the email we should use to contact you.

Email

Powered by Qualtrics
